# Supplementary material for: Development of a Fully Automated Desktop Analyzer and Ultrahigh Sensitivity Digital Immunoassay for SARS-CoV-2 Nucleocapsid Antigen Detection
Source: Biomedicines. 2022 Sep 15;10(9):2291. doi: 10.3390/biomedicines10092291 (PMC9496537; doi:10.3390/biomedicines10092291)
Supplement: Supplementary file 1 [file biomedicines-10-02291-s001.zip › biomedicines-1875449-supplementary.pdf]

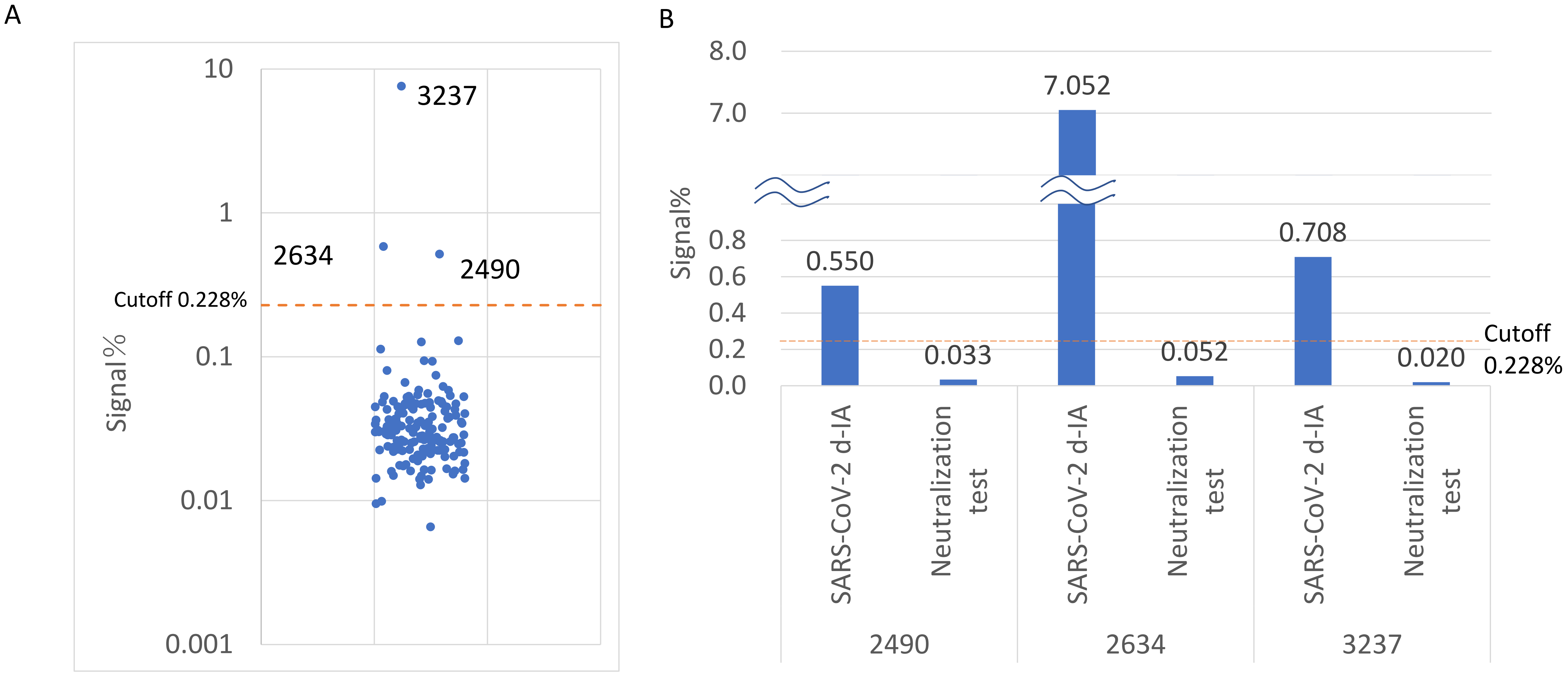

Figure S1. RT-PCR negative distribution, cutoff setting, and neutralization test for three RT-PCR negative/SARS-CoV-2 Ag positive specimens. (A) A Ddot blot of RT-PCR negative swabs results of SARS-CoV-2 d-IA. (B) Results of SARS-CoV-2 d-IA and the neutralization test with 100 nM anti-SARS-CoV-2 nucleocapsid protein antibodies for three specimens over the cutoff (ID: 2490, 2634, and 3237).
